# From rash to spine: A rare case report of Herpes zoster myelitis

**DOI:** 10.1097/MD.0000000000047130

**Published:** 2026-01-23

**Authors:** Meng Zhou, Xia Luo, Jinglun Li

**Affiliations:** a Department of Neurology, The Affiliated Hospital of Southwest Medical University, Luzhou, China; b Laboratory of Neurological Diseases and Brain Function, The Affiliated Hospital of Southwest Medical University, Luzhou, China.

**Keywords:** Herpes zoster myelitis, myelitis, varicella-zoster virus

## Abstract

**Rationale:**

Herpes zoster myelitis is a rare but serious neurological condition. The present case emphasizes the importance of carefully inquiring about the patient’s medical history and conducting a comprehensive physical assessment. Reports of such cases are valuable for raising awareness of herpes zoster complications, reducing misdiagnosis, and guiding clinical treatment.

**Patient concerns:**

A 45-year-old male patient presented with weakness in the left lower limb for more than 20 days, along with a herpetic rash and pain in the left lumbar and left abdominal regions. The patient reported a history of herpes development in June 2024.

**Diagnoses:**

The physical examination upon admission had shown weakness and hypesthesia in the left lower limb. Furthermore, laboratory tests had shown positive IgG antibodies against the varicella-zoster virus in the cerebrospinal fluid. Magnetic resonance imaging of the spinal cord had revealed hyperintense signals on T2-weighted images at the T7–T9 vertebral level. The final diagnosis was herpes zoster myelitis based on clinical and imaging findings.

**Interventions:**

The patient received acyclovir treatment and steroid pulse therapy, which resulted in partial improvement of symptoms.

**Outcomes:**

The patient’s muscle strength improved from 3/5 at admission to 4/5 at discharge; additionally, numbness has decreased, and pain and temperature sensations have improved.

**Lessons:**

The early recognition of rare diseases is paramount, which is not only benefits individual patients but also contributes to accumulating clinical knowledge that enhances collective diagnostic acumen in managing these challenging conditions.

## 1. Introduction

Varicella-zoster virus (VZV) is a neurotropic double-stranded DNA virus belonging to the α-herpesvirus subfamily of the Herpesviridae family. It only infects humans. Herpes zoster is a disease caused by VZV. It typically presents as blisters and pain in local skin areas such as the thoracic and lumbar regions or the head and facial areas.^[1]^ In 1965, Hope-Simpson first proposed the hypothesis that VZV remains latent in sensory ganglia after primary infection. This hypothesis was later confirmed by histological studies. Later scholars discovered the virus in the sensory ganglia of the peripheral nervous system. The virus that is latent in the ganglia can reactivate when the human immune system is weakened.^[2]^ The reactivation of varicella-zoster virus, culminating in herpes zoster, is primarily driven by a decline in virus-specific cell-mediated immunity. Advancing age constitutes the most significant risk factor due to immunosenescence.^[3]^ Furthermore, a spectrum of immunosuppressive conditions, including hematological malignancies, HIV infection, and iatrogenic immunosuppression (e.g., chemotherapy and corticosteroids), substantially elevates the risk.^[4]^ Emerging evidence also implicates certain comorbidities, such as diabetes and autoimmune diseases, as independent predisposing factors, even in seemingly immunocompetent individuals.^[5]^ Herpes zoster typically presents as a self-limiting, localized cutaneous eruption confined to a single dermatome, such as the thoracic, lumbar, or trigeminal regions. The characteristic rash does not cross the midline and usually resolves within 2-4 weeks, with few complications.^[6]^In more severe cases, however, the disease may progress to disseminated zoster. This form is uncommon, occurring in < 5% of immunocompetent individuals but rising to 10% to 20% in immunocompromised hosts. It is defined by a widespread vesicular eruption affecting noncontiguous dermatomes or presenting with a generalized varicella-like rash. Disseminated disease is frequently associated with severe visceral involvement, including the central nervous system (e.g., myelitis, encephalitis), lungs, and liver, necessitating urgent medical intervention.^[7]^ Herpes zoster myelitis (HZM) is one of the rarer types. Through in-depth analysis of this case, we hope to provide a reference for similar cases. We also encourage further discussion on rare complications of herpes zoster to enhance comprehensive understanding of the disease and reduce treatment delays caused by insufficient awareness.

## 2. Case presentation

**Figure 1.**
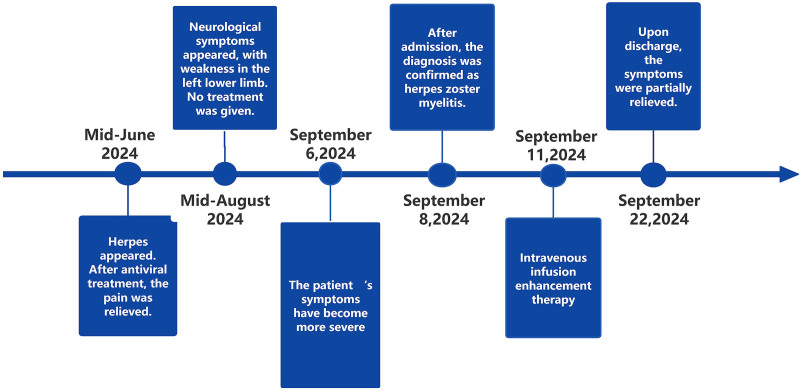
Timeline of disease course.

The patient, a 45-year-old male, was admitted to the hospital on September 8, 2024, with the chief complaint of weakness in the left lower extremity for over 20 days, and the disease course is illustrated in Figure 1. In mid-June 2024, the patient gradually developed a herpes zoster rash on the left lumbar and abdominal area, accompanied by pain. After antiviral treatment at a local hospital, the pain improved and the rash gradually dried up and crusted over. In mid-August 2024, the patient experienced weakness in the left lower extremity, manifested as difficulty climbing slopes and stairs. There was no obvious dragging when walking, and no numbness or discomfort in the limbs. The patient visited a local hospital and underwent a head CT scan, which showed no significant abnormalities, and was discharged without further treatment. After discharge, the patient noted persistent symptoms and subsequently visited our hospital on September 6, 2024. At that time, the main symptoms were difficulty in lifting the left lower extremity independently, dragging when walking, and numbness in the left lower extremity. Nerve conduction velocity tests of both lower extremities showed decreased motor conduction velocity of the left tibial nerve, low amplitude of compound muscle action potential, and decreased sensory conduction velocity. The motor conduction amplitude of the left common peroneal nerve was low, and the sensory conduction velocity was decreased. No abnormalities were found in the right tibial nerve, and common peroneal nerve. The outpatient physician considered the diagnosis to be “herpes zoster combined with peripheral neuropathy” and prescribed valacyclovir 0.3 g, twice daily; prednisone acetate 20 mg, once daily; and aluminum magnesium carbonate chewable tablets 1 g, 3 times daily. Despite taking the prescribed medications, the symptoms did not improve significantly, leading to the patient’s readmission on September 8, 2024, for further treatment. The patient has a history of pulmonary tuberculosis, which he reports having been cured.

**Figure 2.**
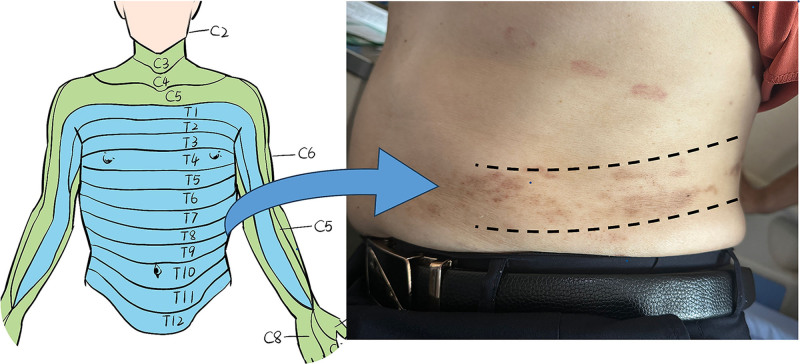
When the patient was admitted, scattered annular plaques and areas of hyperpigmentation were observed in the left abdominal region, corresponding to the T9 spinal nerve root dermatome.

Physical examination upon admission revealed a body temperature of 36.7°C, pulse rate of 80 beats per minute, respiratory rate of 20 breaths per minute, and blood pressure of 123/78 mm Hg. Scattered ring-shaped scabs and pigmentation were observed on the left flank(Approximately at T9) (Fig. 2). No significant abnormalities were found on general internal examination. The neurological examination showed the patient was conscious with fluent speech and normal higher cortical function. Cranial nerve examination was unremarkable. Muscle strength was 5/5 in the left upper limb, 3/5 in the left lower limb, and 5/5 in the right upper limb, with normal muscle tone in all limbs. Tendon reflexes were normal. Sensory testing revealed decreased pain sensation in the left lower extremity, while deep sensation was intact. Ankle clonus was present on the left side, and pathological reflexes were absent bilaterally. The patient exhibited an steppage gait.

**Figure 3.**
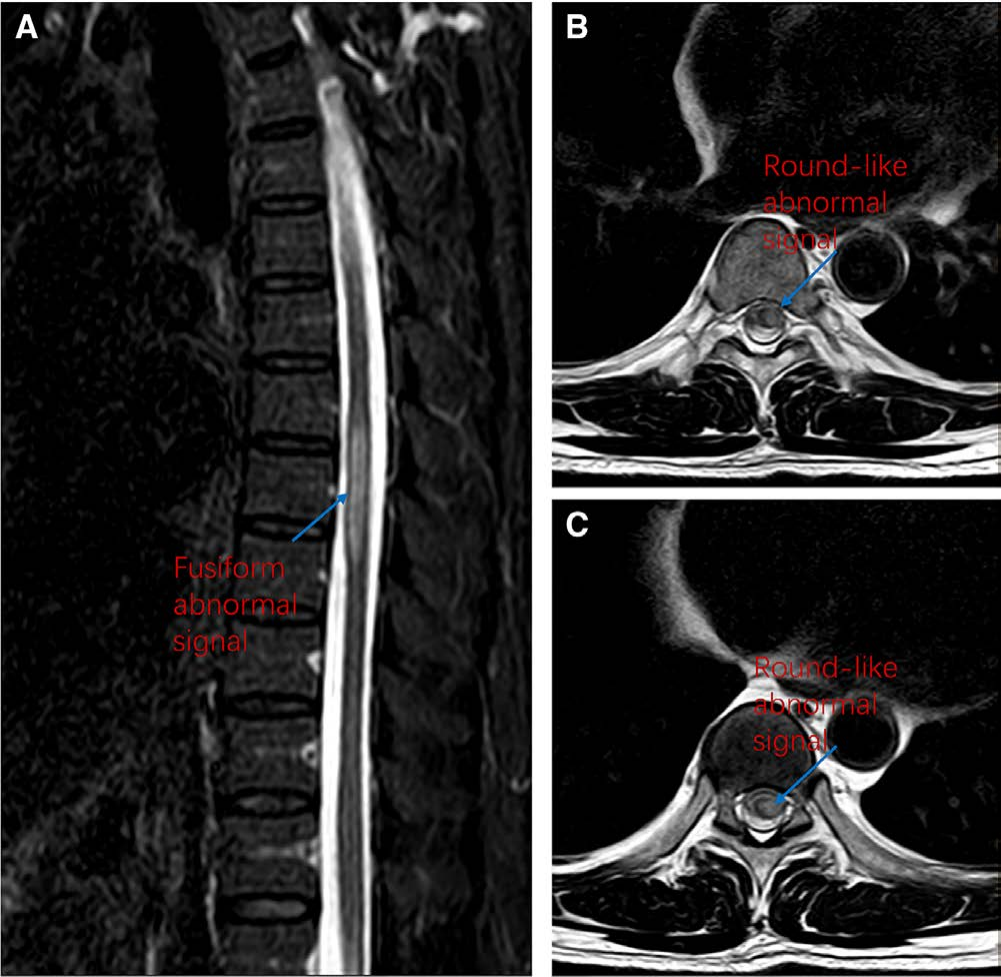
The sagittal plane shows T2 fat-suppressed sequence, with slightly high signal shadows at T7 to T9. (A) The cross-section is T2 sequence, showing a round-like low signal shadow at T8. (B) The cross-section is T2 sequence, showing a round-like low signal shadow at T9 (C).

Auxiliary examinations: Cerebrospinal fluid routine showed that the color was colorless, clarity was clear and transparent, and no clot formation was observed. The protein qualitative test was negative. The white blood cell count was 11 × 10^6 cells/L, with 91% mononuclear cells and 9% polymorphonuclear cells. The red blood cell count was 0 × 10^6 cells/L, and red blood cell morphology showed no red blood cells observed. Cerebrospinal fluid biochemistry revealed the following: lactate dehydrogenase, 31.4 U/L; glucose, 3.34 mmol/L; lactate, 1.95 mmol/L; chloride, 123.5 mmol/L; and protein, 0.432 g/L. Varicella-zoster virus IgG (VZV-IgG) was positively detected in cerebrospinal fluid. Targeted high-throughput sequencing (tNGS) of cerebrospinal fluid yielded a negative result. Spinal cord magnetic resonance imaging showed a fusiform abnormal signal at the T7–9 vertebral level of the spinal cord (Fig. 3). The lesion measured approximately 0.7 cm × 0.6 cm × 4.0 cm in size. It was isointense on T1WI and exhibited high signal on T2WI and FLAIR, with clear boundaries. Nerve conduction velocity studies of both lower extremities revealed decreased motor conduction velocity of the left tibial nerve with low amplitude, and decreased sensory conduction velocity. The left common peroneal nerve showed low amplitude of motor conduction and decreased sensory conduction velocity. No abnormalities were detected in the right tibial nerve and common peroneal nerve.

Treatment plan: After admission, antiviral treatment with acyclovir (0.5 g, every 8 hours, 9-day course) was initiated. Based on preventive anti-tuberculosis treatment with rifampicin (0.45 g, once daily) and isoniazid (0.3 g, once daily), a 3-day steroid pulse therapy at 0.5 g once daily was administered.

Prognosis and outcome: The patient’s muscle strength improved from 3/5 at admission to 4/5 at discharge; additionally, numbness has decreased, and pain and temperature sensations have improved.

## 3. Discussion

After the herpes virus invades the spinal cord, the virus causes direct damage by replicating in target cells and causing their lysis and necrosis. It also causes indirect damage by inducing immune responses and mediating immune inflammation. This includes VZV infection of endothelial cells, which causes vascular inflammation, vascular wall necrosis, and thrombosis. Additionally, viral antigens activate T cells (mainly CD8 + cytotoxic cells) and macrophages, which infiltrate the spinal cord parenchyma in large numbers. These immune cells promote the release of numerous inflammatory factors, exacerbating tissue edema, necrosis, and demyelination. Moreover, viral antigens (such as glycoproteins) exhibit molecular mimicry with host neural antigens, inducing cross-reactive antibodies and T cells that attack the myelin sheath and neurons.^[8,9]^ As shown in Table 1, apart from the varicella-zoster virus, a number of other viruses have been implicated as rare causes of acute transverse myelitis, with distinct virological characteristics and varying clinical outcomes.,^[10–13]^ In this case, the patient presented with left lower limb weakness and superficial sensory disturbance as the main manifestations. These symptoms were mostly due to damage to the anterior horn motor neurons or demyelination and/or necrosis of the corticospinal tract and spinothalamic tract caused by viral infection, representing a focal neurological insult. In essence, when host immunity is severely compromised, primary or reactivated VZV can disseminate hematogenously. This is often accompanied by a generalized varicella-like rash, systemic manifestations such as high fever and chills, and visceral infection manifestations such as esophagitis, hepatitis, and pneumonia, posing an immediate life-threatening risk with high acute-phase mortality.^[14,15]^ In contrast, patients with herpes zoster myelitis, while having lower acute mortality, frequently develop severe and permanent neurological disabilities, including paralysis and chronic bladder/bowel dysfunction, which profoundly impair quality of life. Therefore, early differentiation between these 2 distinct VZV-induced entities is critical for guiding therapeutic intervention and prognostic assessment.

**Table 1:** The similarities and differences between herpes zoster virus and other rare viruses in causing spinal cord inflammation.

Viral Etiology	Typical Host	Key Clinical features	Rate of Complete Recovery	Prognosis & Comparison to VZV
Varicella-Zoster (VZV)	Immunocompromised/Elderly	Often dermatomal rash, vasculopathy	Variable	Moderate, risk of residual deficits and chronic pain.
Hepatitis E (HEV)	Immunocompetent (endemic)	GBS overlap common	High	Generally better than VZV. Often complete recovery.
West Nile Virus (WNV)	All ages	Poliomyelitis-like, acute flaccid paralysis	Low	Generally worse than VZV. High rate of severe disability.
Epstein-Barr (EBV)	Adolescents/Young adults	Mononucleosis symptoms	Moderate	Similar to VZV. Mixed outcomes.
Cytomegalovirus (CMV)	Severely immunocompromised	Aggressive, ascending myelitis	Low (without IRIS)	Worse than VZV. Dependent on immune recovery.
Herpes Simplex-2 (HSV-2)	Immunocompetent	Relapsing course, with meningitis	Variable	Distinct from VZV due to relapsing nature.

GBS = Guillain-Barré syndrome, IRIS = immune reconstitution inflammatory syndrome.

HZM is relatively rare in clinical practice, with an annual incidence of about 0.3%. This rarity is mainly due to the fact that VZV primarily spreads in an anterograde manner along sensory neurons. Additionally, the structural barrier of the spinal cord and the immune surveillance function of glial cells can effectively clear small amounts of virus transmitted in a retrograde manner.^[16]^ Moreover, the clinical manifestations of HZM are diverse and often atypical, with some cases lacking the characteristic rash,^[17]^ which further increases the difficulty of diagnosis. Together, these factors make the invasion of VZV into the spinal cord parenchyma and subsequent myelitis a rare complication. Despite a few reports, it is uncommon for patients with normal immune function to develop myelopathy due to VZV infection.^[18]^ The uniqueness of this case lies in the patient’s past medical history, including treatment for tuberculosis, which may have contributed to immune function impairment. In this patient, weakness in the left lower extremity occurred in mid-August, suggesting the possibility of spinal cord disease, and the prolonged latency between the onset of the rash and the development of neurological deficits, significantly exceeding the typical 1- to 2-week window, suggests a more indolent disease course.^[19]^ Additionally, a skin lesion remained on the left side of the waist, indicating possible complications related to VZV. However, when the patient visited a local hospital, no obvious abnormalities were found on head computed tomography (CT), and no further treatment was administered. Half a month later, the patient came to our hospital for further evaluation. Based on the patient’s condition, a spinal magnetic resonance imaging scan was performed, revealing a fusiform hyperintense signal in the T7-9 segments of the spinal cord spatially corresponding to the skin lesion. This manifestation could be due to herpes zoster myelitis but could also result from demyelinating diseases of the spinal cord, tumor lesions (such as ependymomas, astrocytomas), syringomyelia, and other conditions. The patient’s medical history, clinical manifestations, and imaging examinations all supported the diagnosis of HZM. However, for infectious diseases, the gold standard for a definitive diagnosis is pathogen detection. Therefore, we performed an invasive examination—lumbar puncture. Routine and biochemical tests of cerebrospinal fluid showed a mild increase in white blood cells mainly composed of lymphocytes, a borderline increase in lactate level, and normal biochemical parameters, suggesting the possibility of viral infection. In addition, detection of VZV-IgG in cerebrospinal fluid was positive. At this point, the diagnostic evidence was consolidated, and the diagnosis was nearly confirmed. We also performed targeted high-throughput sequencing (tNGS) on the cerebrospinal fluid. However, due to improper sample collection, low pathogen load, limitations of detection technology, and other factors, we were unable to extract the nucleic acid information of the VZV.^[20]^We then administered antiviral therapy combined with moderate-dose corticosteroid pulse therapy. The patient improved partially and was discharged. Given that the patient’s clinical presentation has not reached complete remission despite a clear diagnosis of HZM, it is necessary to be vigilant about the possibility of concurrent Neuromyelitis optica spectrum disorder (NMOSD). Previous studies have reported cases of co-occurrence of HZM and NMOSD. To exclude the presence of NMOSD, further testing for serum aquaporin-4 antibody (AQP4-IgG) and myelin oligodendrocyte glycoprotein antibody (MOG-IgG) should be conducted. Although the exact pathophysiological relationship between HZM and NMOSD remains unclear and requires confirmation through larger-scale studies, active screening for NMOSD-specific antibodies in patients with a clear diagnosis of HZM, but poor therapeutic response, and concurrent long-segment spinal cord lesions is of significant clinical importance.^[21,22]^This approach aids in clarifying the diagnosis, guiding treatment strategies, and assessing prognosis. Currently, there is no established treatment plan for HZM. In previous cases, combined antiviral and corticosteroid therapy was mostly used, but the optimal hormone dosage remains controversial.^[23]^In this case, corticosteroids were administered primarily to mitigate the inflammatory damage to the spinal cord parenchyma triggered by the viral infection. While antiviral agents halt viral replication, it is often the vigorous and sometimes exaggerated host immune response, manifested as perivascular inflammation, cytotoxic edema, and immune-mediated demyelination, that causes neural injury. By virtue of their potent anti-inflammatory and immunosuppressive properties, corticosteroids attenuate this aberrant cellular immune response. This action alleviates spinal cord edema and may halt the progression of demyelination, thereby potentially facilitating improved neurological recovery.^[24]^ Some scholars have reported the application of interferon, human immunoglobulin, and plasma exchange in the treatment of herpes zoster myelitis, with varying degrees of effectiveness.^[25,26]^The overall prognosis is good.

In summary, the patient’s disease progression and treatment response indicate the potential risks of the varicella-zoster virus in immunocompromised patients and the complexity of its clinical manifestations. Clinicians should enhance their understanding of VZV; promptly conduct cerebrospinal fluid examinations, and imaging examinations; and optimize treatment plans. People with weakened immune systems are more prone to reactivation of VZV and severe neurological complications. Therefore, we advocate that individuals with compromised immunity or those preparing for immunotherapy^[27]^ should receive the herpes zoster vaccine whenever possible to reduce the occurrence of adverse clinical outcomes.

## 4. Conclusions

Herpes zoster myelitis, though infrequent, carries significant risks of long-term neurological sequelae, including motor deficits, sensory disturbances, and chronic pain, which can profoundly impact patient quality of life. The current absence of consensus on key management aspects, such as optimal antiviral dosing, timing and duration of immunomodulatory therapy, and criteria for monitoring treatment response. Establishing clear therapeutic standards will enhance clinical consistency, improve patient outcomes, and provide a framework for future research to refine management strategies for herpes zoster myelitis.

## Acknowledgments

The authors would like to thank our department colleagues and the patient for his dedication. This was a fully anonymized, retrospective study for which the requirement for ethical review was waived by the Institutional Review Board (IRB) as informed consent had been obtained from the patient.

## Author contributions

**Data curation:** Meng Zhou, Xia Luo.

**Visualization:** Xia Luo, Jinglun Li.

**Writing – original draft:** Meng Zhou.

**Writing – review & editing:** Meng Zhou, Xia Luo, Jinglun Li.
